# A Hand-Worn Inertial Measurement Unit for Detection of Bat–Ball Impact during Baseball Hitting

**DOI:** 10.3390/s21093002

**Published:** 2021-04-25

**Authors:** Niroshan G. Punchihewa, Hideki Arakawa, Etsuo Chosa, Go Yamako

**Affiliations:** 1 Department of Materials and Informatics, Interdisciplinary Graduate School of Agriculture and Engineering, University of Miyazaki, Miyazaki 889-2192, Japan; jb17007@student.miyazaki-u.ac.jp; 2 Department of Rehabilitation, Faculty of Medicine, University of Miyazaki, Miyazaki 889-2192, Japan; hideki_arakawa@med.miyazaki-u.ac.jp; 3 Department of Orthopaedics, Faculty of Medicine, University of Miyazaki, Miyazaki 889-2192, Japan; chosa@med.miyazaki-u.ac.jp; 4 Department of Mechanical Engineering, Faculty of Engineering, University of Miyazaki, Miyazaki 889-2192, Japan

**Keywords:** batting, kinematics, IMU, acceleration, accuracy

## Abstract

Swinging a baseball bat at a pitched ball takes less than half of a second. A hitter uses his lower extremities to generate power, and coordination of the swing motion gradually transfers power through the trunk to the upper extremities during bat–ball impact. The most important instant of the baseball swing is at the bat–ball impact, after which the direction, speed, height, and distance of the hit ball determines whether runs can be scored. Thus, analyzing the biomechanical parameters at the bat–ball impact is useful for evaluating player performance. Different motion-capture systems use different methods to identify bat–ball impact. However, the level of accuracy to detect bat–ball impact is not well documented. The study aim was to examine the required accuracy to detect bat–ball impact timing. The results revealed that ±2 ms accuracy is required to report trunk and hand kinematics, especially for higher-order time-derivatives. Here, we propose a new method using a hand-worn inertial measurement unit to accurately detect bat–ball impact timing. The results of this study will be beneficial for analyzing the kinematics of baseball hitting under real-game conditions.

## 1. Introduction

Baseball is an outdoor sport in which the team that scores the most runs within nine innings wins the game. Hitters try to hit a baseball thrown by a pitcher from the opposite team as hard as they can so that the ball travels a long distance or is directed with sufficient accuracy and ball-exit velocity (BEV) that the fielder either cannot get to the ball or makes a fielding error. Either case will allow the baserunner(s) on offense to advance through the bases and score runs. The BEV at the bat–ball impact is a critical performance measurement of a baseball hitter.

The baseball bat-swing motion has been analyzed by using optical motion-capture systems (OMCS) and force plates to understand the kinematic and kinetic parameters [1,2,3]. However, it is difficult to set up the OMCS in a real-game environment, so the majority of studies have been conducted in laboratory settings and involve hitting a stationary baseball off a tee-pole [4,5,6]. Recent developments of inertial measurement units (IMUs) have allowed researchers to investigate human motion in a real-life environment [7,8]. The validity and reliability of IMUs for capturing baseball swings have been recently reported [9,10].

The timing at the bat–ball impact during baseball hitting needs to be captured closely to investigate important movement parameters and quantify the bat speed and BEV. When using an OMCS, the timing of the bat–ball impact is generally monitored by high-speed cameras or the movement of reflective markers attached to the baseball and bat [11,12,13]. The ball is in contact with the bat for <1.5 ms [14]. Thus, the timing of the bat–ball impact is generally considered to be the first frame before the collision [15,16,17]. The actual timing of the impact will deviate by a maximum of 5 ms when data is captured at a 200 Hz sampling rate. The impact time captured by IMUs with a 200 Hz sampling rate has been reported to give an accuracy of 9 ms in tee–bat settings [18]. However, the variability of the biomechanical parameters with the deviation of impact timing is unknown, so it is difficult to validate the accuracy of the timing of the bat–ball impact reported previously. The purpose of this study was to examine the variability in the kinematic parameters when the impact time deviates from the actual timing. We propose a new method to accurately detect bat–ball impact timing that uses an IMU with a sampling rate of 1000 Hz attached to a hand. Wooden bats are used in professional baseball leagues, whereas aluminum bats are used by high-schools and amateur baseball players. The performance of wooden and aluminum bats differ significantly [19]. Thus, both bat types were used in this study to evaluate the proposed method.

## 2. Materials and Methods

### 2.1. Participants

Five male baseball players (four right-handed, one left-handed, age: 20.0 ± 0.7 years; height: 1.7 ± 0.1 m; weight: 72.4 ± 10.0 kg) who were members of the Miyazaki University Baseball Club voluntarily participated in the study. None of the participants had a known history of musculoskeletal or neurological diseases. The study protocol was approved by the ethics committee of the university, and written informed consent was obtained from each participant prior to data collection.

### 2.2. Instrumental Setup

**Figure 1:**
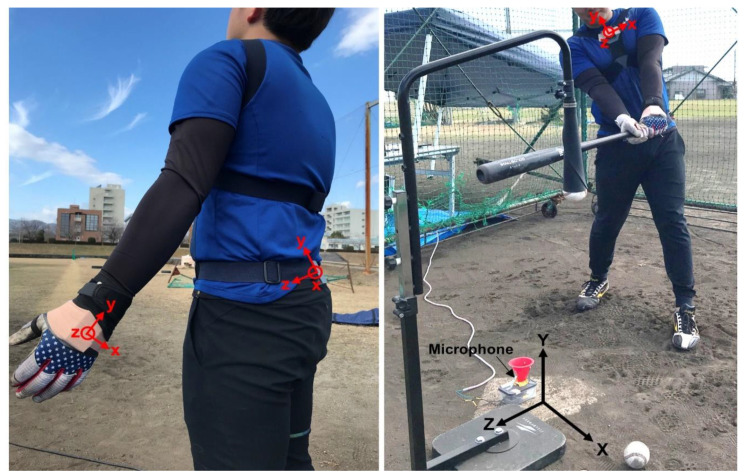
IMU placement and the experimental setup at the tee hitting session. Upper-case letters (X, Y, Z) denote field coordinates, whereas lower-case letters (x, y, z) denote local axes in each IMU attached to the pelvis, thorax, and knob-side hand.

Three IMUs (sampling rate of 1000 Hz, SS-MS-HMA200G60 (accelerometer (1962 ms^−2^), gyroscope (6000° s^−1^), magnetometer (10 gauss)), size: 36 mm (width) × 53 mm (length) × 11 mm (depth), weight: 32 g; Sports Sensing Co., Ltd., Fukuoka, Japan) were attached to body segments of the players prior to data collection. One IMU was attached to the superior part of the sternum, and a second IMU was attached between the left and right iliac spines by using a 3D-printed attachment and elasticized tapes (Figure 1). Both IMUs were attached such that the *x*-axis was parallel to the medio–lateral direction toward the left side, and the *y*-axis was directed upwards along the longitudinal axis according to anatomical landmarks of the corresponding segments. The third IMU was attached to the dorsal side of the knob-side hand by using double-sided tape over the batting glove and further secured with an elasticized bandage. The positive *y*-axis of the local coordinates was aligned with the long axis toward the proximal direction, and the *z*-axis was directed outwards toward the hand. Data captured by each IMU were stored inside the internal memory. 

Fully charged IMUs were kept for 10 min after switching on to allow the IMUs to reach a steady-state temperature. The accelerometer and magnetometer were calibrated before the IMUs were attached to the body. A similar calibration procedure was used as described in our previous study [10].

### 2.3. Data Collection during In-Field Hitting

The experiment was conducted at an outdoor baseball field. The hitting measurements were divided into two sessions. In each session, the players used an aluminum bat and a wooden bat to hit a baseball. Players used their personal wooden bat, but the same aluminum bat was used by all players throughout the trials. The players completed a self-selected warm-up period before the hitting experiment. In the first session, the players hit a baseball hanging on a tee five times, with an interval of ≥30 s between each swing. The ball height was adjusted approximately to the waist height when the player was in his hitting stance. A safety net was used to trap the batted balls. In the second session, the players hit a baseball pitched by a machine. The ball height at home plate was approximately adjusted to the waist height, and the ball speed was set to the regular practice speed of the baseball club. The session was continued until the player was able to hit five balls (as line drives) toward center field. All trials in which the bat contacted the ball were recorded. The players rested in-between the trials to reduce fatigue.

Before each measurement, the players were instructed to stand still for 5 s while facing home plate and keeping the hand straight (*y*-axis of the attached IMU was vertically upwards) alongside the body (the player’s sagittal plane was parallel to a line drawn between the pitching plate and home plate), which allowed calculation of the gyroscope offset and initial orientation of each IMU. After all the trials were completed, IMU data were transferred to a computer for data processing. IMU data processing was completed by using a script written in MATLAB (Version 2017b, MathWorks^®^, Natick, MA, USA).

### 2.4. Calculation of the Trunk and Hand Kinematics

The orientation of each IMU was calculated in the form of a unit quaternion (*q*) by using a gradient-descent fusion algorithm [20]. A similar approach presented in a previous study was adopted to calculate IMU orientation [10]. The orientation of the pelvis IMU during the static pose and the gravity vector were used to define new ground coordinates (Figure 1) aligned with the baseball field [21]. The orientation of each IMU was then calculated with respect to the new ground coordinates. Acceleration and angular velocity vectors registered in the local coordinates of each IMU were transformed into ground coordinates. Acceleration and angular velocity data were low-pass filtered (cutoff frequency = 12 Hz) using a fourth-order Butterworth filter, and linear velocity was calculated by numerically integrating the acceleration data. Kinematic parameters from 0.8 s before the impact to 0.2 s after the bat–ball impact were used for further analysis. The kinematic data of a left-handed player were mirrored in accordance with a right-handed player.

### 2.5. Impact-Detection Algorithm

**Figure 2:**
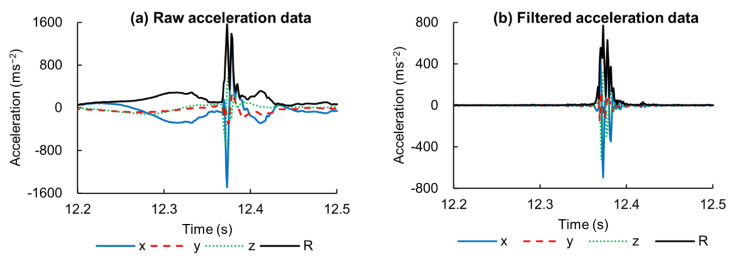
Hand acceleration when hitting a baseball (**a**) before and (**b**) after data was filtered through a high-pass Butterworth filter (cutoff = 100 Hz).

Accelerometer data of the hand IMU was used to detect the impact time. The detected time was compared with the time retrieved by a microphone described in the next section. Some energy is dispersed along the bat in the form of vibration after bat–ball impact and is heavily damped at the hands [22]. This could be detected by the IMU acceleration attached to the hand. Hand acceleration consists of three major components: acceleration due to swing motion, the gravity vector, and acceleration due to vibration (Figure 2a). Swing motion and gravity are categorized as having low-range frequency. Accelerometer data were filtered by using a fourth-order (zero-lag), high-pass Butterworth filter with a cutoff frequency of 100 Hz to isolate the vibration signal (Figure 2b).

**Figure 3:**
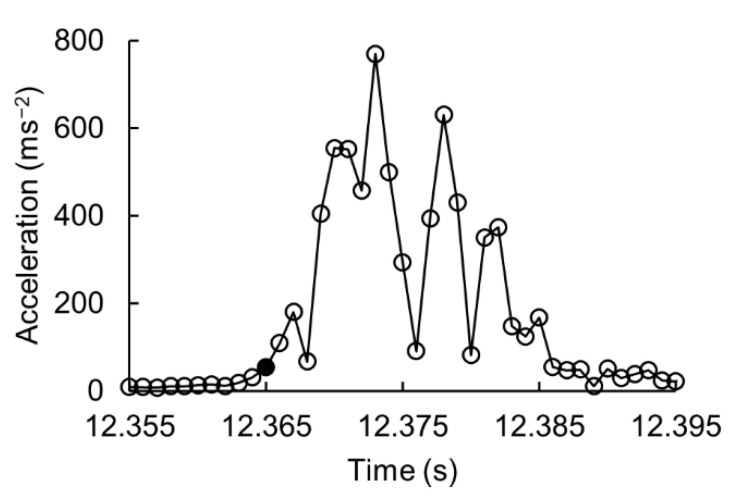
Resultant acceleration calculated from the high-pass filtered (cutoff = 100 Hz) hand-acceleration data. Solid marker • denotes the impact time identified by the microphone.

The ball–bat impact could be seen just after the rise of the resultant acceleration (Figure 3). However, neither the maximum value was closer nor a threshold value could be easily determined. Thus, we used acceleration in each axis, and the local maxima was used to detect the impact point. The complete algorithm is described as follows: 

**Step 1:** Hand acceleration is high-pass filtered (a fourth-order Butterworth filter) with a cutoff frequency = 100 Hz (Figure 2b).

**Step 2:** Acceleration difference of the adjacent data points are calculated in each axis.

**Figure 4:**
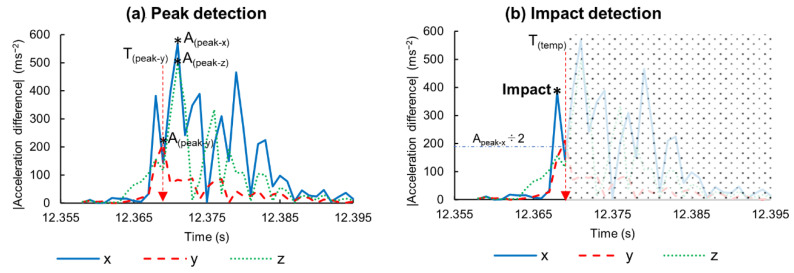
Absolute acceleration difference calculated from the high-pass filtered acceleration data of the hand IMU. (**a**) Peak values and the corresponding times detected from absolute acceleration difference (Step 3). (**b**) Local maxima greater than that of the half of the corresponding peak value (Step 5). The shaded area is excluded from the calculation after the first peak identification.

**Step 3:** Absolute differences are taken, and the peak value of each axis (A _(peak-x)_, A _(peak-y)_, A _(peak-z)_) and respective times (T _(peak-x)_, T _(peak-y)_, T _(peak-z)_) are obtained (Figure 4a).

**Step 4:** The earliest time is selected as a temporary time stamp (T_temp_ = T _(peak-y)_) in this case).

**Step 5:** Acceleration differences are limited up to T_temp_, and the times at the first local maxima higher than that of A_peak_ ÷ 2 are examined in the corresponding axes (Figure 4b).

**Step 6:** If the impact time exists, the earliest time is taken as the impact time; otherwise, T_temp_ is used as the impact time.

### 2.6. Impact-Time Validation

Impact time detected by the hand IMU was compared with the time retrieved by a microphone (Module KY-037) placed on home plate (Figure 1). The microphone was powered by direct current at 5V and connected with wires to a data acquisition system (DAQ) (NR 600, Keyence Cooperation, Osaka, Japan). The DAQ was switched on for 30 min to warm-up, and necessary calibration was performed before data collection. The DAQ was connected to a computer via a USB cable, and data were stored in each trial. A 5V pulse trigger was used to synchronize the IMUs and the DAQ.

**Figure 5:**
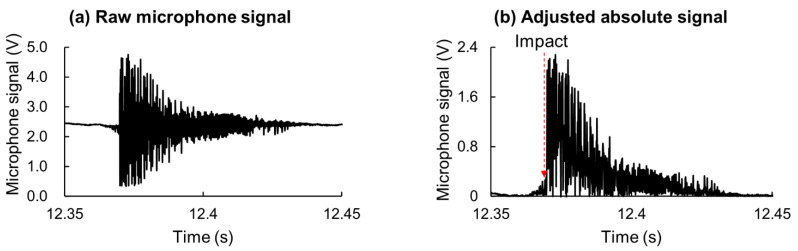
Bat–ball impact sound detected by the microphone as (**a**) raw signal and (**b**) adjusted signal to capture impact time.

The impact sound detected by the microphone data was stored as a voltage difference at a 10-KHz sampling rate. The offset voltage was subtracted from the microphone data, and initiation of the voltage spike was detected as the impact time (Figure 5). The time lag due to the distance between the microphone and impact location was calculated by using the waist height and sound velocity (343 ms^−1^).

### 2.7. Statistical Analysis

A measurement was considered successful when both the IMU and DAQ systems recorded valid data and an audible bat–ball contact was made. We used 157 successful trials for the analysis (68 trials from the single aluminum bat (tee = 25, pitched = 43) and 89 trials from the wooden bats (tee = 25, pitched = 64)). The deviation of the trunk and hand kinematics from their corresponding values at impact were calculated for ±10 ms to define the maximum agreement of the error. Only tee-batting trials were considered to reduce intra-player variation. The mean deviation of the segmental angles, angular velocities, linear velocities, and linear accelerations were taken for the evaluation. Since different kinematic parameters use different units, the deviation was calculated as the mean absolute error (MAE) represented as a percentage of the corresponding absolute peak value. An MAE of <5% was considered to be excellent agreement, and between 5% and 10% was considered to be an acceptable measurement.

The error of the impact timing was calculated as the mean time difference, standard deviation (SD), and root mean square error (RMSE) to assess the accuracy of the proposed algorithm. The homogeneity of variance was calculated between hitting sessions (tee vs. pitched) for each bat type (aluminum vs. wood) by using Levene’s test. Two-way analysis of variance was used to evaluate any significant difference between bat types or the hitting sessions to detect the bat–ball impact. The level of significance was set to 0.05. Data were analyzed by using Statistical Package for the Social Sciences software (Version 22.0, IBM Corp., Tokyo, Japan).

## 3. Results

### 3.1. Deviation of the Kinematic Parameters from Impact

**Figure 6:**
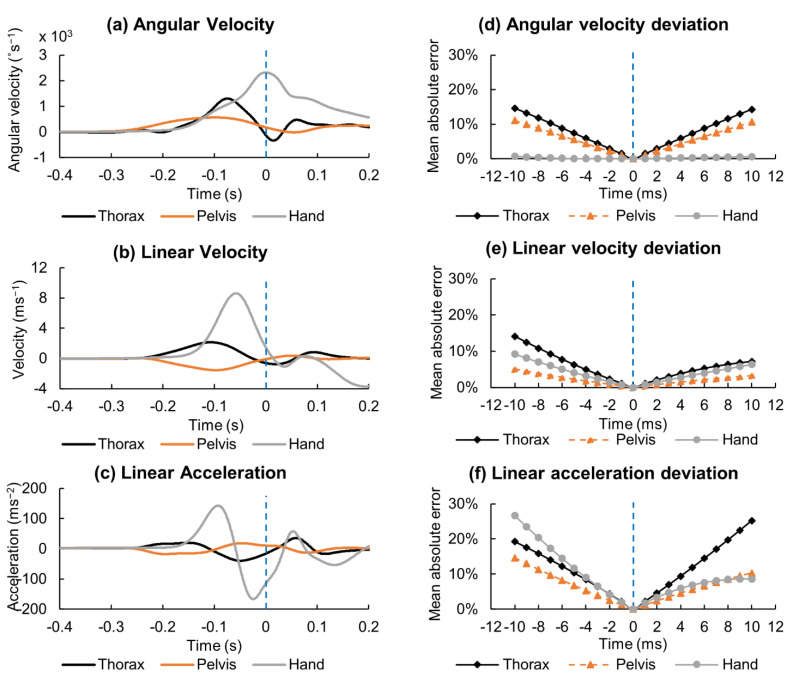
Typical graphs of (**a**) angular velocity in *Y*-axis, (**b**) linear velocity and (**c**) linear acceleration of *X*-axis of the thorax, pelvis, and hand segments. Deviation of the (**d**) angular velocity, (**e**) linear velocity, and (**f**) linear acceleration from the value at the bat–ball impact within ±10 ms represented as the mean absolute error as a percentage of the corresponding absolute peak value. Vertical broken line indicates the bat-ball impact.

The MAE of angular velocity, linear velocity, and linear acceleration of the thorax, pelvis, and hand segments were calculated for ±10 ms from the impact time. Segmental angular velocities in the vertical axis (*Y*-axis), linear velocity, and acceleration toward the pitcher’s direction (*X*-axis) are plotted in Figure 6a–c and corresponding MAE are plotted in Figure 6d–f respectively. Angular velocity of the hand reached its peak at bat–ball impact. Thus, MAE of the hand angular velocity calculated as a percentage of the corresponding peak was almost zero. Both angular velocity and linear velocity errors reached ≥10% beyond ±7 ms in one segment at least, whereas the error was <5% within ±3 ms. The linear acceleration error exceeded 10% beyond ±4 ms, which required the impact time to be detected within ±2 ms to reduce the error to <5%. The MAEs of the thorax and pelvis angles were ≤10% within ±10 ms and so are not included in the figures.

### 3.2. Impact-Time Error

The time difference detected by the hand IMU and the microphone at the bat–ball impact were assessed separately for aluminum and wooden bats in each session and presented as the mean error, SD, and RMSE in Table 1. Both the mean error and RMSE were consistent at 1 ms, with the maximum SD of 1 ms for a tee-batting session using a wooden bat.

Levene statistics confirmed the equality of variances among hitting sessions for the aluminum bat (F = 0.202, *p* = 0.65) and for each wooden bat (F = 1.489, *p* = 0.226). There were no statistically significant differences in the mean error between bat types (*p* = 0.466) and hitting sessions (*p* = 0.300).

## 4. Discussion

Baseball hitting is a highly dynamic activity. Players use both lower and upper extremities to generate energy to increase the bat speed and transfer momentum to achieve higher BEV at the bat–ball impact. Thus, it is important to break down the biomechanical description of baseball hitting in a timely manner and to accurately detect the bat–ball impact time to evaluate player performance. The study results revealed that the selected kinematic parameters varied as much as 20% when the timing of the bat–ball impact deviated ±10 ms from the actual timing. Our proposed method detected the impact time within 1 ms of error, indicating excellent accuracy for identifying bat–ball impact by using a hand-worn IMU. 

### 4.1. Accuracy of Bat–Ball Impact Timing

The MAEs of the orientation angles of the trunk segments differed by <10% within ±10 ms. However, the linear and angular velocities required to capture the impact time within ±7 ms for kinematic variables to be acceptable (MAE < 10%) and within ±3 ms were expected to accurately measure (MAE < 5%) the kinematic variables. Linear acceleration further limited the accuracy to ±2 ms for excellent agreement. Therefore, high-speed cameras and OMCS require capturing biomechanical parameters at a ≥500 Hz sampling rate to accurately monitor higher-order derivatives at the bat–ball impact.

**Figure 7:**
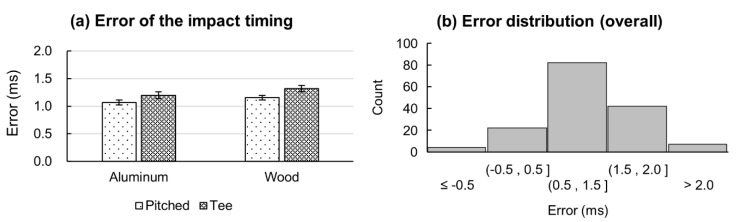
(**a**) Impact timing error in tee and pitched hitting conditions in each bat type and (**b**) overall error distribution.

Figure 7a shows that the impact error is slightly less in pitched hitting than in stationary-baseball hitting. A bat is in contact for a shorter period of time during high-speed collisions (pitched) than during low-speed collisions (tee) [23]. Energy is dispersed through the bat faster in the form of vibration and thus, the propagated signal could be detected at the end of the bat quicker for shorter collision times than for longer collision times. Additionally, when the bat collides with a baseball, the peak reaction force occurs earlier in aluminum bats than in wooden bats [24]. However, the time difference between groups was <1 ms, which is the smallest reliable measurement when sampling at 1000 Hz. Therefore, no significant difference could be measured between the bat types or hitting sessions.

The errors of the impact timing detected by a hand IMU showed an approximately normal distribution centered at 1 ms (Figure 7b). The overall error distribution demonstrated that the method introduced in this study had minimal systematic errors for detecting bat–ball impact [25].

### 4.2. Hand Acceleration Caused by Impact Force

Vibration magnitudes vary with grip firmness and impact location [26]. Acceleration of each axis showed different amplitudes in each trial depending on the impact location and contact angle. The first peak was not necessarily the highest peak on each axis due to different frequency modes, and so required a second iteration to closely detect the impact time. Furthermore, the acceleration magnitude was not consistent for each bat–ball contact. Therefore, a common threshold value could not be determined to detect the first local peak. Thus, the acceleration difference was calculated, and half of the highest peak was used as the threshold value to determine the impact time.

The dynamics of the baseball swing could be calculated if the IMU was fixed on the bat knob [27]. However, impact vibration exceeded the accelerometer measurement range (1962 ms^−2^) during the initial testing, which could damage the accelerometer. Further, some players felt discomfort when gripping the bat near the knob, so the IMU was attached to the dorsal side of the hand.

### 4.3. Limitations

Only the trunk and hand kinematic parameters were calculated to assess the variability at the bat–ball impact. A baseball-hitting motion starts at the lower extremities, and coordination of the motion is gradually transferred to the upper extremities during bat–ball impact. Thus, trunk and hand kinematics are well-selected parameters for evaluation. However, the variation of the bat head speed around the impact time should be further evaluated.

We assumed that the ball was hit directly over the microphone at waist height, but the spatial margin of error may have affected detection of the impact time in the pitched-ball hitting sessions. Nevertheless, the impact detected by the algorithm had an error range between −1 ms and 3 ms.

## 5. Conclusions

The bat–ball impact time needs to be detected within ±2 ms to accurately monitor higher-order trunk and hand kinematics in baseball hitting. We suggest using ≥500 Hz frame rates for baseball-hitting analysis when using an OMCS or high-speed cameras. IMUs have recently shown promising accuracy for baseball-hitting analysis. The new method using a hand-worn IMU at a 1000 Hz sampling rate introduced in this study accurately detected bat–ball impact times. 

## Figures and Tables

**Table 1:** Mean difference, standard deviation (SD), and root mean square error (RMSE) of the impact time detected by the microphone and hand IMU for each bat type in each hitting session.

Bat Type	Hitting Session	Mean (SD)	RMSE
Aluminum	Pitched	1.1 (±0.8)	1.3
	Tee	1.2 (±0.6)	1.4
	All	1.1 (±0.7)	1.3
Wood	Pitched	1.2 (±0.8)	1.4
	Tee	1.3 (±1.0)	1.6
	All	1.2 (±0.9)	1.5

Units are in milliseconds.

## Data Availability

Not applicable.
